# The effects of mindfulness enhanced Tai Chi Chuan training on mental and physical health among beginners: a randomized controlled trial

**DOI:** 10.3389/fpsyg.2024.1381009

**Published:** 2024-09-06

**Authors:** Ping Qu, Xiaoqing Zhu, Hui Zhou, Zhengyu Kang, Ran Li, Jingsi Wen, Feng Pan, Yang Liu, Ting Zhu, Qian Cao, Xiaoyan Wang, Yuyin Wang

**Affiliations:** ^1^Department of Physical Education, Sun Yat-Sen University, Guangzhou, China; ^2^Department of Psychology, Sun Yat-Sen University, Guangzhou, China; ^3^Department of Physical Education, Guangdong Technical Normal University, Guangzhou, China; ^4^Shandong Institute of Physical Education, Shandong, China

**Keywords:** Tai Chi Chuan, mindfulness, mental health, physical health, beginners

## Abstract

**Introduction:**

Tai Chi Chuan (TCC) is a traditional Chinese mind–body exercise widely adopted in Chinese communities and colleges. However, the mindful essence of TCC is rarely emphasized during popularization. This makes it difficult for beginners to benefit from it. The present study aimed to examine the effects of a Mindfulness-enhanced Tai Chi Chuan (MTCC) intervention, which enhances mindfulness components embedded within TCC, on mental and physical health among beginners.

**Methods:**

A randomized controlled trial was conducted with 119 healthy college students new to Tai Chi Chuan training. Participants were assigned to either the MTCC group or the TCC group. Both interventions consisted of 10 weekly 90-min training sessions, with the MTCC group emphasizing and enhancing mindfulness components. Outcome measures included mindfulness, depression, anxiety, stress, and physical fitness, assessed at baseline and post-intervention.

**Results:**

The results showed that the MTCC group had significantly greater improvements than the TCC group in mindfulness, anxiety, stress, and health—and skill-related physical fitness. There were no significant differences between the two groups in depression.

**Conclusion:**

The findings suggest that compared to TCC, MTCC can effectively promote individuals’ physical fitness and provide additional benefits to mental well-being. MTCC can be recommended as an accessible and beneficial intervention for beginners to improve mental health and strengthen their bodies.

**Clinical review registration:**

https://www.chictr.org.cn/, identifier ChiCTR2200058175.

## 1 Introduction

Tai Chi Chuan (TCC) is a traditional Chinese exercise that aims to promote the coordination of the body, mind, and spirit through a slow sequence of movements (e.g., deep breathing and pushing hands). TCC is also regarded as a mindfulness-based exercise emphasizing physical balance and mental concentration to maintain an attentive presence (La Forge, 2016). Currently, TCC is being promoted in many Chinese communities as an aerobic exercise with mild-to-moderate intensity. Existing studies have proved the beneficial effects of TTC for people of all ages, but only small-to-moderate effects on mental health were observed (see Yin et al., 2023, for a meta-analysis). This could be because some training programs teach TCC as a purely physical exercise regimen and do not emphasize the mindful aspect of the process (Henning et al., 2021). Simply imitating movement without understanding TTC from the perspective of meditation and mindfulness will weaken its health benefits, especially for beginners of TCC. Therefore, we developed a mindfulness-enhanced Tai Chi Chuan (MTCC) intervention, which involves traditional TTC training with a unique emphasis on mindfulness, to maximize the benefits of TTC for beginners.

TCC is a form of mild-to-moderate aerobic activity often recommended for the elderly to attenuate the decline of physical and psychological functions (Leung et al., 2022). However, TCC may also provide an exercise alternative for young adults who live a sedentary lifestyle and are at risk of preventable diseases but lack the motivation to engage in more conventional exercise (Lin et al., 2022). Additionally, TCC can help individuals attain a meditative state of mind, which allows them to concentrate on the present moment and counteract daily stresses by activating the parasympathetic nervous system (Yeung et al., 2018). This can be particularly beneficial for young adults who are at risk of experiencing chronic stress and the resulting higher likelihood of developing mental health problems. A systematic review including 9,263 participants from 76 studies proved that TCC is likely to benefit college students by increasing flexibility, reducing symptoms of depression, decreasing anxiety, and improving interpersonal sensitivity (Webster et al., 2016). Considering these benefits, more and more Chinese universities have begun offering TCC training programs as part of physical education courses.

However, previous studies on the effects of TCC among young adults have produced conflicting results, particularly regarding its mental health benefits. For instance, one RCT reported no significant improvement in psychological outcomes such as mindfulness, stress, and mood among college students after TCC exercise (Zheng et al., 2015). Research also revealed that the effectiveness of TCC was not as good as other mind–body exercises such as Yoga, Mindfulness, and Yi Jin Jing (Hua and Sun, 2021; Shen et al., 2018). A possible explanation for these unsatisfactory results could be that some TCC programs focus primarily on practicing movements, relatively neglecting the importance of the mindfulness component. Mindfulness embedded within TCC emphasizes a profound inward mental focus over and above the physical movements, distinguishing TCC from conventional exercises such as running and swimming (La Forge, 2016). However, during the traditional TCC training, the instructor usually did not verbally introduce or periodically guide “mindfulness.” Consequently, it may be challenging for young beginners to understand the connotations of TCC from the perspective of mindfulness and psychologically benefit from it.

Researchers have investigated the health benefits of mindfulness-based Tai Chi Chuan in physical and mental health (Zhang et al., 2018; Zhang et al., 2021). The mindfulness-based Tai Chi Chuan involves modified exercise forms mixed mindfulness and simplified 24 short-form TCC, mainly focused on present, non-judgment, peaceful state involving TCC movements (Zhang et al., 2018). However, we believe that simply combining mindfulness skills with traditional TCC forms may not be enough. It is also essential to consider enhancing mindfulness components during the arrangement of TCC forms. Based on this idea, we developed a 16-form Taiyi Mirrored-heart Chuan with a unique emphasis on mindfulness and further formed a complete Mindfulness-enhanced Tai Chi Chuan (MTCC) intervention program.

Therefore, this study aimed to examine the effects of the Mindfulness-enhanced Tai Chi Chuan intervention for mental health and physical fitness in young beginners with a randomized controlled trial (RCT) design. We hypothesized that the intervention would increase participants’ mindfulness and physical fitness while reducing psychological distress (i.e., depression, anxiety, and stress).

## 2 Materials and methods

### 2.1 Participants

**Figure 1:**
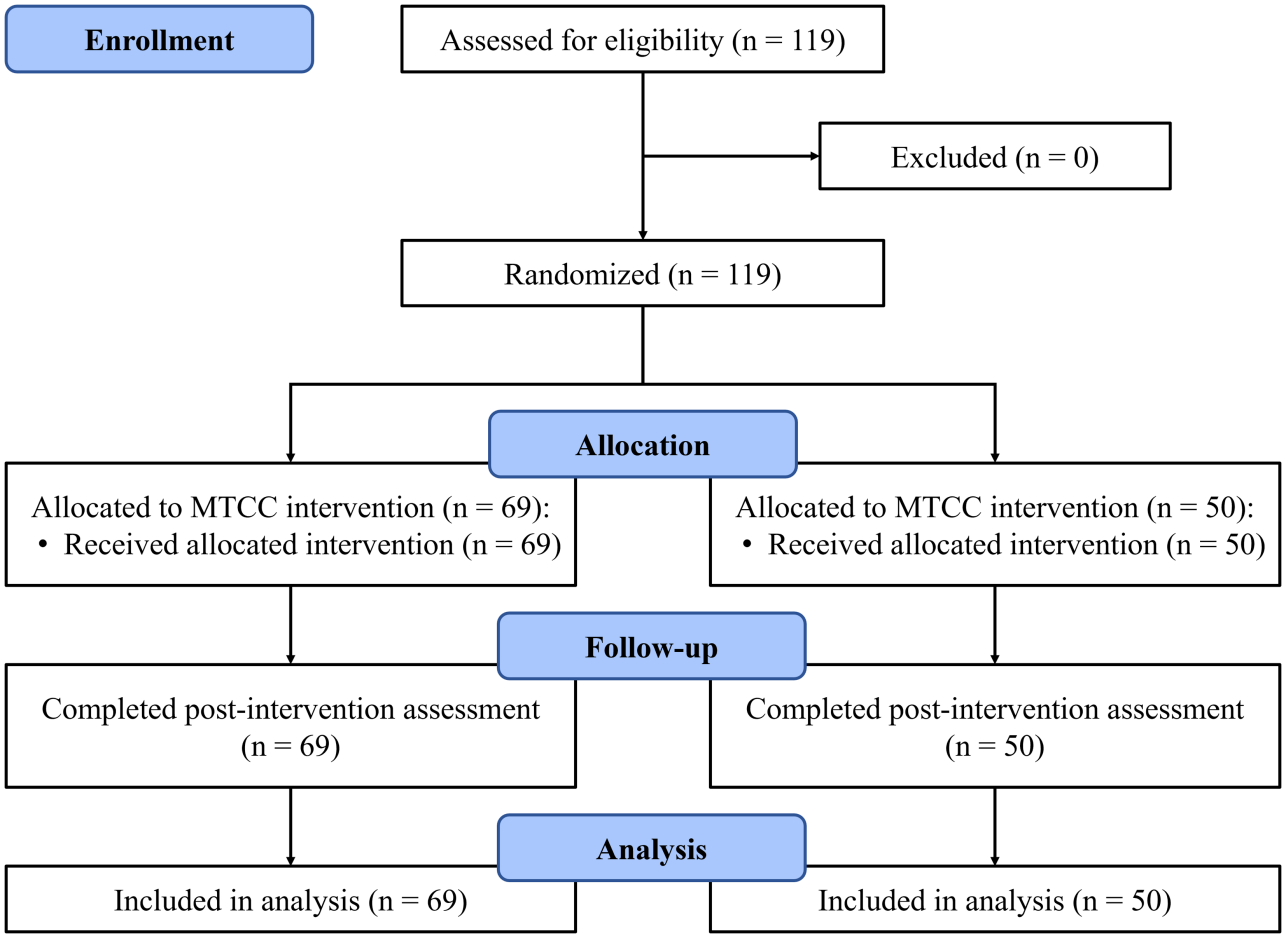
CONSORT diagram.

We conducted a prior power analysis using G*Power (Faul et al., 2007) for a 2 (between-subject factors: MTCC group vs. TCC group) × 2 (within-s factors: baseline vs. post-intervention) mixed ANOVA and found that a sample size of 46 would be needed to detect a medium effect size (Cohen’s *f* = 0.25, power = 0.80, correlation among repeated measures = 0.30). As illustrated by Figure 1, we recruited 119 healthy college students from Sun Yat-Sen University, Guangzhou, China. The inclusion criteria were as follows: (1) Aged 17–22 years; (2) No history of physiological or psychological disorders; (3) No contraindications to exercise; (4) No smoking or drinking habits; (5) No previous experience with mind–body techniques (e.g., Tai Chi Chuan, yoga, and meditation). All participants provided informed consent to participate in the study. Then they were randomly assigned to receive Mindfulness-enhanced Tai Chi Chuan (MTCC) intervention or traditional Tai Chi Chuan (TCC) intervention. All participants completed the study. The final sample consisted of 69 participants in the MTCC group (18 males and 51 females; mean age = 18.06, *SD* = 0.51) and 50 participants in the TCC group (10 males and 40 females; mean age = 18.14, *SD* = 0.61).

### 2.2 Procedure

Tai Chi Chuan is a physical education course for all undergraduates at Sun Yat-Sen University, and students who took this course were eligible to participate. Limited by the course arrangement and classroom place, we set up two classes (maximum of 35 students, respectively) teaching MTCC, and one class (maximum of 50 students) teaching TCC. 119 eligible participants were assigned to one of the three classes using a computer-generated randomization table. Both interventions consisted of 10 weekly 90-min training sessions led by qualified university physical education instructors. All participants completed the assessments of mental health and physical functioning approximately 1 week before the intervention (T1) and within 1 week after the intervention (T2). Approval of the study was obtained from the Ethical Committee of the Department of Psychology at Sun Yat-Sen University. The study was registered on https://www.chictr.org.cn/ (ID: ChiCTR2200058175). To reduce participants’ burden from excessively long questionnaire completion times, we removed the secondary outcome measure of self-esteem mentioned in the registered protocol prior to data collection.

### 2.3 Interventions

The Mindfulness-enhanced Tai Chi Chuan (MTCC) involved training in a 16-form Taiyi Mirrored-heart Chuan developed by the research team. The training was adapted from 24-form Yang-style Tai Chi Chuan, Yi Jin Jing (Sinew-transforming Qigong Exercises), and Tai Chi Tui Shou (Pushing Hands), with a unique emphasis on mindfulness. Over the 10 weeks of the intervention, participants met for 90 min once a week in a classroom on the campus. During the intervention, instructors introduced information about Taiyi Mirrored-heart Chuan and mindfulness and guided participants through a series of practices. Participants were also encouraged to do home practices following the guidelines provided by instructors.

The first session provided an introduction to Taiyi Mirrored-heart Chuan and mindfulness. The following sessions advanced participants’ Tai Chi Chuan skills and understanding of mindfulness through several trainings and practices. Each session consisted of a 25-min review and preparation, 45-min Taiyi Mirrored-heart Chuan training, 10-min mindfulness-based practices, and a 10-min ending part for summarizing and assigning homework. It should be noted that mindfulness is embedded within each session. The instructors would remind participants to maintain mindful awareness and acceptance throughout the learning and practicing. More details about the MTCC intervention are presented in the Supplementary material.

The traditional Tai Chi Chuan (TCC) involved training in the 24-form Yang-style Tai Chi Chuan, widely recognized by the General Administration of Sport of China. TCC had a program structure and duration similar to MTCC’s. The main distinction between the interventions was the extent to which mindfulness was emphasized. During the TCC training, instructors did not explicitly introduce mindfulness or instruct participants to approach Tai Chi Chuan with a mindfulness-focused mindset.

### 2.4 Measures

#### 2.4.1 Mindfulness

The 15-item Mindful Attention Awareness Scale (MAAS) (Brown and Ryan, 2003) was used to assess individual differences in the frequency of attention to and awareness of present-moment experiences (e.g., “I find myself doing things without paying attention”). Participants rated on a 6-point Likert scale from 1 (almost always) to 6 (almost never). The average score was calculated after reverse-scoring negatively worded items, with higher scores indicating a greater level of dispositional mindfulness. The Chinese version of this measure was validated in previous studies (Chen et al., 2012). The Cronbach’s alpha in this study were 0.87 (T1) and 0.86 (T2).

#### 2.4.2 Depression, anxiety, and stress

The 21-item Depression Anxiety Stress Scale (DASS) (Lovibond and Lovibond, 1995) was used to measure the emotional states of depression (e.g., “I felt that I had nothing to look forward to”), anxiety (e.g., “I felt I was close to panic”), and stress (e.g., “I found it hard to wind down”) over the past week. Participants rated on a 4-point Likert scale from 0 (did not apply to me at all) to 3 (applied to me very much or most of the time). The average score was calculated within each subscale, with higher scores indicating higher levels of depression, anxiety, and stress. The Chinese version of this measure was validated in previous studies (Gong et al., 2010). The Cronbach’s alpha of the three subscales in this study were 0.78 (T1) and 0.78 (T2) for depression, 0.70 (T1) and 0.68 (T2) for anxiety, 0.80 (T1) and 0.75 (T2) for stress, respectively.

#### 2.4.3 Physical fitness

Physical fitness tests were used as indicators to assess participants’ general physical health. Health-related physical fitness was measured by (1) Body Mass Index (BMI; body composition), (2) push-ups (upper limb strength and endurance), (3) abdominal crunch (abdominal strength and endurance), (4) sit and reach (flexibility), and (5) endurance running (endurance). Skill-related physical fitness was measured by (1) one-leg stand (balance), (2) standing long jump (lower limb explosive strength), (3) 50-m running (speed), (4) ruler drop (reaction time), and (5) 1-min rope skipping (coordination). The scores of health-related and skill-related physical fitness were calculated separately using a composite point based on China’s National Student Physical Health Standard (Ministry of Education of the People’s Republic of China, 2014). Specifically, an overall score for each aspect of physical health was calculated using Z-score and T-score conversions for each domain (Weger et al., 2012).

### 2.5 Data analyses

Independent *t*-tests or chi-square tests were conducted to examine the baseline differences between the MTCC group and the TCC group. Next, a 2 (group: MTCC vs. TCC) × 2 (time: baseline vs. post-intervention) repeated-measures analysis of variance (ANOVA) was conducted to compare the intervention effect between two groups on outcome measurements. When the interaction effect of time × group was significant, a *post hoc* comparison was conducted to determine the statistical significance of differences between and within groups over time. The effect sizes were indicated by partial eta squared (*η*^2^; 0.01: small, 0.06: moderate, 0.14: large; Cohen, 1988). All statistical analyses were performed using IBM SPSS 27.

## 3 Results

There were no significant baseline differences between the MTCC group and the TCC group in demographic characteristics or study variables (*p* > 0.05). Table 1 displays the descriptive statistics of the study variables for the two groups.

**Table 1:** Baseline characteristics of the study sample.

Characteristics	MTCC group (*n* = 69)	TCC group (*n* = 50)	*t*/χ^2^	*p*
Age	18.06 ± 0.51	18.14 ± 0.61	0.799	0.426
Gender, *n* (%)			0.597	0.440
Male	18 (26.1%)	10 (20%)		
Female	51 (73.9%)	40 (80%)		
Mindfulness	4.07 ± 0.72	4.22 ± 0.74	1.114	0.268
Depression	1.52 ± 0.37	1.38 ± 0.39	−1.899	0.060
Anxiety	1.66 ± 0.35	1.56 ± 0.38	−1.482	0.141
Stress	1.83 ± 0.49	1.67 ± 0.50	−1.721	0.088
Health-related physical fitness	138.77 ± 13.01	134.21 ± 15.53	−1.738	0.085
Skill-related physical fitness	238.09 ± 30.20	237.98 ± 24.58	−0.022	0.982

**Figure 2:**
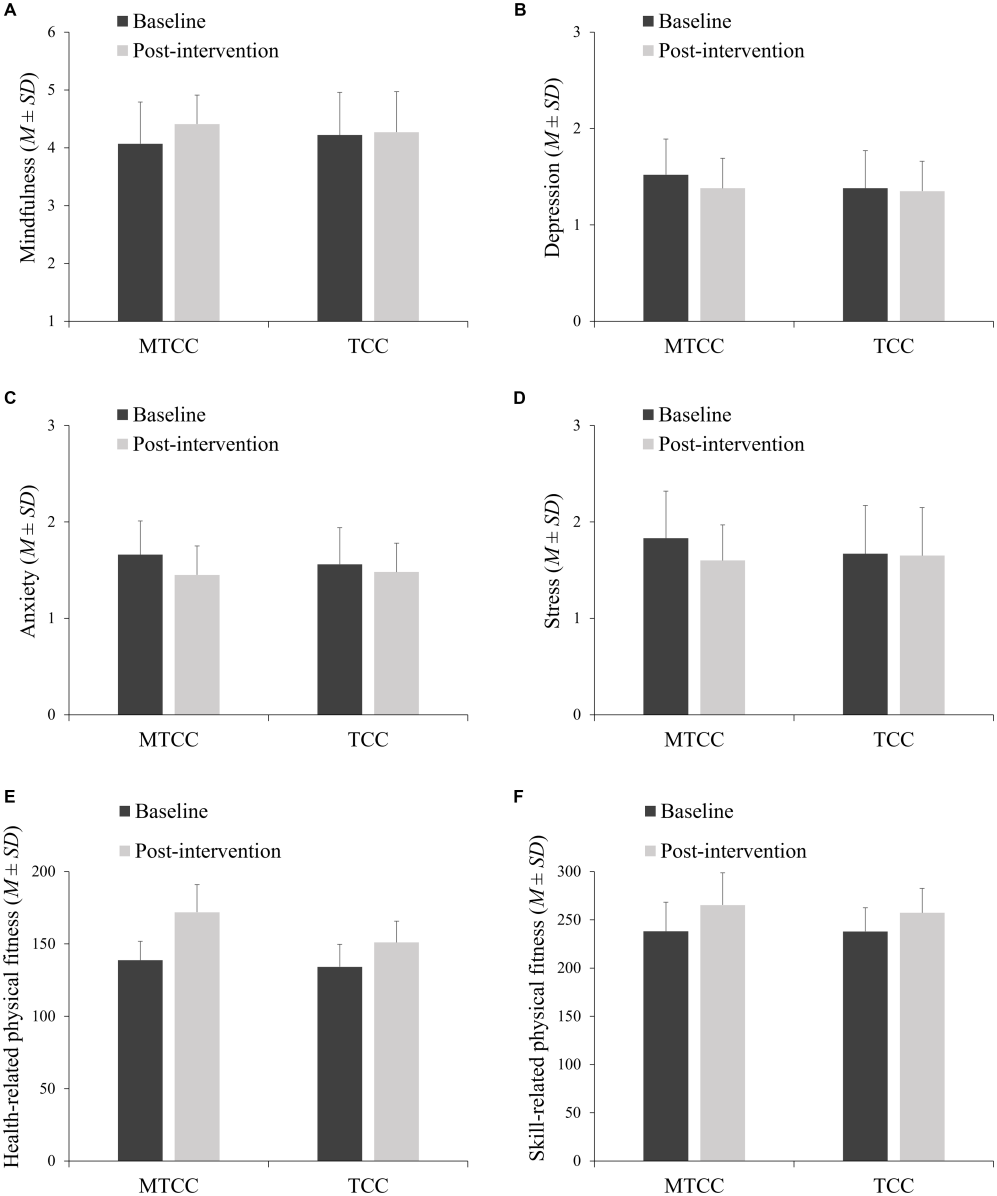
Comparisons of effects of two groups before and after intervention.

As shown in Table 2 and Figure 2, repeated-measures ANOVA indicated significant interactions between time and group in mindfulness (*F* (1, 117) = 4.038, *p* = 0.047, *η*^2^ = 0.033), anxiety (*F* (1, 117) = 4.640, *p* = 0.033, *η*^2^ = 0.038), and stress (*F* (1, 117) = 6.191, *p* = 0.014, *η*^2^ = 0.050). Small to moderate effect sizes were observed for these outcomes. There was no significant interaction in depression (*F* (1, 117) = 2.779, *p* = 0.098, *η*^2^ = 0.023). Within-group simple effect analysis showed that for the MTCC group, the mean scores in mindfulness (*F* (1, 117) = 13.224, *p* < 0.001, *η*^2^ = 0.102) increased, as well as depression (*F* (1, 117) = 12.149, *p* < 0.001, *η*^2^ = 0.094), anxiety (*F* (1, 117) = 31.965, *p* < 0.001, *η*^2^ = 0.215) and stress (*F* (1, 98) = 17.845, *p* < 0.001, *η*^2^ = 0.132) decreased significantly after the intervention in the MTCC group. However, there were no significant changes in the TCC group (*p* > 0.05).

**Table 2:** Results of repeated-measures ANOVA.

	MTCC group (*n* = 69)		TCC group (*n* = 50)		*F*	*p*	*η* ^2^
	Baseline	Post-intervention	Baseline	Post-intervention			
Mindfulness	4.07 ± 0.72	4.41 ± 0.50	4.22 ± 0.74	4.27 ± 0.70	4.038	0.047	0.033
Depression	1.52 ± 0.37	1.38 ± 0.31	1.38 ± 0.39	1.35 ± 0.31	2.779	0.098	0.023
Anxiety	1.66 ± 0.35	1.45 ± 0.30	1.56 ± 0.38	1.48 ± 0.30	4.640	0.033	0.038
Stress	1.83 ± 0.49	1.60 ± 0.37	1.67 ± 0.50	1.65 ± 0.50	6.191	0.014	0.050
Health-related physical fitness	138.77 ± 13.01	171.91 ± 19.13	134.21 ± 15.53	151.05 ± 14.76	31.089	< 0.001	0.210
Skill-related physical fitness	238.09 ± 30.20	265.33 ± 33.39	237.98 ± 24.58	257.29 ± 25.41	9.530	0.003	0.075

Regarding the intervention effect on physical fitness, repeated measures ANOVA showed a significant interaction effect between time and group in health-related physical fitness (*F* (1, 117) = 31.089, *p* < 0.001, *η*^2^ = 0.210) and skill-related physical fitness (*F* (1, 117) = 9.530, *p* = 0.003, *η*^2^ = 0.075). Moderate to large effect sizes were observed for these outcomes. Within-group simple effect analysis showed that scores for health-related physical fitness increased significantly after intervention in both the MTCC group (*F* (1, 117) = 305.719, *p* < 0.001, *η*^2^ = 0.723) and the TCC group (*F* (1, 117) = 57.179, *p* < 0.001, *η*^2^ = 0.328). Similarly, scores for skill-related physical fitness increased significantly after intervention in both the MTCC group (*F* (1, 117) = 268.118, *p* < 0.001, *η*^2^ = 0.696) and the TCC group (*F* (1, 117) = 97.707, *p* < 0.001, *η*^2^ = 0.455).

## 4 Discussion

This study aimed to examine the effects of a 10-week Mindfulness-enhanced Tai Chi Chuan (MTCC) intervention on enhancing mental health and physical fitness among beginners. To our knowledge, this is the first attempt to optimize the benefits of TCC for young beginners by incorporating and enhancing mindfulness components during the training. The results indicated that, compared to TCC, MTCC significantly improved participants’ health—and skill-related physical fitness. Additionally, participants who received MTCC showed significant improvement in mindfulness, and reductions in stress and anxiety.

Compared to traditional Tai Chi Chuan, one prominent feature of MTCC was the emphasis on a mindful state when practicing movements. In the present study, this enhancement has proven successful as participants who practiced MTCC demonstrated significantly greater improvements in health-and skill-related physical fitness. A meta-analysis on mindfulness-based interventions have found small to moderate effect sizes of mindfulness-based interventions for physical conditions (Crowe et al., 2016). The moderate to large effect sizes observed in the present study suggest that MTCC holds promise as an effective intervention for promoting physical fitness in the college student population. These additional benefits are likely due to the mindfulness-enhanced components, which increase awareness and attention to body movements and sensations during MTCC practice (Wimmer et al., 2020). This heightened awareness, in turn, may facilitate the learning and performance of complex and coordinated movements, contributing to physical fitness gains.

Moreover, the MTCC program has proven to be more than just a physical fitness program. The results showed that MTCC have small to moderate effects on improving mindfulness while reducing anxiety and stress among beginners, which is consistent with effect sizes reported in meta-analyses of mindfulness-based interventions (Dawson et al., 2019; Galante et al., 2023). Given the pervasive nature of mental health challenges, these effect sizes, although not large, underscore the practical significance of MTCC as a potential tool for addressing psychological distress among young adults. The beneficial effects of MTCC on mental health are driven by the embedded mindfulness components, which cultivate the awareness and acceptance of one’s thoughts, emotions, and bodily sensations (Treves et al., 2019). This enhanced mind state reduces the negative appraisal and rumination of stressful events (Michl et al., 2013) and equips individuals with skills for managing stress and promoting emotional resilience (Galante et al., 2018). Additionally, MTCC integrated the physical, mental, and spiritual aspects of Tai Chi Chuan with mindful awareness, which may have synergistic effects on psychological well-being (Si et al., 2020).

Inconsistent with our hypotheses, we did not find significant changes in depression from baseline to post-intervention in the MTCC group compared to the TCC group. One possible reason for this unexpected finding is that the depression levels in our sample were relatively low, leaving little room for further improvement. It is also worth noting that previous studies have typically used a frequency of twice a week (Zhang et al., 2018) or three times a week (Chen et al., 2021) to ensure training effectiveness. Our weekly intervention may not have been sufficient to produce clinically significant changes, especially for students with mild to moderate depressive symptoms. However, we did find trends that participants in the MTCC group showed more reduction in depression from baseline to post-intervention. This result also suggests that we may consider further optimizing the intervention program to enhance the effect.

### 4.1 Limitations and future research

Although we use a well-controlled randomized trial design, this study also has some limitations that should be acknowledged. First, due to the use of convenience sample of college students, caution is advised when generalizing the results. Second, the predetermined course arrangement limited the number of participants assigned to the two groups. However, no significant baseline differences were found between the two groups, indicating that the random assignment was successful. Third, we only measured the outcomes immediately after the intervention, which may not reflect the long-term effects of MTCC on beginners’ mental health and physical fitness. Future research should include follow-up assessments to examine the maintenance and durability of the health benefits over time.

Beyond the physical and mental health benefits, MTCC may offer several unique advantages for beginners that deserve further attention (e.g., accessibility and learning efficacy). Specifically, MTCC’s emphasis on mindfulness fosters a welcoming and supportive learning environment for beginners (Kerrigan et al., 2017). The focus on introspection and non-judgmental awareness reduces the pressure to perform perfectly, making it more accessible for beginners with varying levels of physical ability or prior experience. Moreover, mindfulness promotes focus and attention to detail, allowing beginners to better understand and execute the movements more precisely (Zhang et al., 2016). This more profound engagement with the practice can lead to faster skill acquisition and improved form, ultimately enhancing the learning experience.

## 5 Conclusion

In conclusion, this study proved that a 10-week mindfulness-enhanced Tai Chi Chuan (MTCC) intervention effectively promotes physical fitness and mental health in beginners. These findings suggest that MTCC, which emphasizes the mindful essence of Tai Chi Chuan, offers unique advantages for beginners to improve their understanding of Tai Chi Chuan, and promote physical and mental well-being. It can be recommended as a more accessible and effective intervention for individuals new to Tai Chi Chuan. In future studies, follow-up assessments, multimodal data collection, and mechanism exploration will be needed to further optimize the MTCC program.

## Data Availability

The raw data supporting the conclusions of this article will be made available by the authors, without undue reservation.
