# Supplementary material for: The effects of mindfulness enhanced Tai Chi Chuan training on mental and physical health among beginners: a randomized controlled trial
Source: Front Psychol. 2024 Sep 6;15:1381009. doi: 10.3389/fpsyg.2024.1381009 (PMC11413537; doi:10.3389/fpsyg.2024.1381009)
Supplement: Supplementary file 1 [file Table_1.DOCX]

Supplementary Material

# Supplementary Table 1. Mindfulness-enhanced Tai Chi Chuan intervention content.

|  | **Major Contents** | **Home Practice** | **Reading Materials** |
| --- | --- | --- | --- |
| **Session 1** | 1. Introduction 2. Mindfulness practice: Mindful Breathing | 1. Mindfulness practice: Mindful Breathing | 1. Introduction to Taiyi Mirror Heart Chuan 2. What is Mindfulness |
| **Session 2** | 1. Review & Discussion 2. Taiyi Mirror Heart Chuan: Form 0−2    - Preparation: Take three deep breaths    - Starting Posture (起势)    - Lifting up and Standing (顶天立地式) 3. Mindfulness practice: Awareness of Body Sensations | 1. Taiyi Mirror Heart Chuan practice 2. Mindfulness practice: Mindfulness Activities in Daily Life | 1. Challenges and Coping Strategies in Tai Chi Chuan Practice 2. The Story of Yang Luchan, the Founder of Yang-Style Tai Chi Chuan |
| **Session 3** | 1. Review & Discussion 2. Taiyi Mirror Heart Chuan: Form 3−4    - Three Plates Falling on the Floor (三盘落地式)    - Grasp the Bird’s Tail Left-hand (左揽雀尾) 3. Mindfulness practice: Body Scan | 1. Taiyi Mirror Heart Chuan practice 2. Mindfulness practice: Body Scan | 1. Observing Your Own Bodily Responses 2. Body Scan |
| **Session 4** | 1. Review & Discussion 2. Taiyi Mirror Heart Chuan: Form 5−6    - Needle at Sea Bottom Left-hand (左海底针)    - Flashing the Arm Left-hand (左闪通臂) 3. Mindfulness practice: Mindfulness of Pleasant Events | 1. Taiyi Mirror Heart Chuan practice 2. Mindfulness practice: Pleasant Events Calendar | 1. Seizing the Moment 2. The Story of Nick Vujicic |
| **Session 5** | 1. Review & Discussion 2. Taiyi Mirror Heart Chuan: Form 7−9    - Grasp the Bird’s Tail Right-hand (右揽雀尾)    - Needle at Sea Bottom Right-hand (右海底针)    - Flashing the Arm Right-hand (右闪通臂) 3. Mindfulness practice: Loving Kindness Meditation | 1. Taiyi Mirror Heart Chuan practice 2. Mindfulness practice: Unpleasant Events Calendar, Loving Kindness Meditation | 1. Caring for Yourself |
| **Session 6** | 1. Review & Discussion 2. Taiyi Mirror Heart Chuan: Form 10 & Review    - Forearm Rollings Both Sides (左右倒卷肱) 3. Mindfulness practice: Breathing Space | 1. Taiyi Mirror Heart Chuan practice 2. Mindfulness practice: Unpleasant Events Calendar; Breathing Space | 1. Breathing Space |
| **Session 7** | 1. Review & Discussion 2. Taiyi Mirror Heart Chuan: Form 11−12    - Single Pushing Hand on Flat Round (平圆单推手)    - Single Pushing Hand of Fold (折叠单推手) 3. Mindfulness practice: Living with Stress | 1. Taiyi Mirror Heart Chuan practice 2. Coping daily stress with mindfulness practices (Body Scan, Loving Kindness Meditation, Breathing Space) | 1. Introduction to Tai Chi Push Hands 2. The Open Mind |
| **Session 8** | 1. Review & Discussion 2. Taiyi Mirror Heart Chuan: Form 13-14    - Withdrawing the Step and Rollback (大捋)    - Prostrating and Looking Upward (昂首仰天式) 3. Mindfulness practice: Cultivate Gratitude Heart | 1. Taiyi Mirror Heart Chuan practice 2. Mindfulness practice: Daily recording what you are grateful for | 1. The Life Wisdom in Tai Chi Chuan 2. A Grateful Heart |
| **Session 9** | 1. Review & Discussion 2. Taiyi Mirror Heart Chuan: Form 15−16    - Swinging the Tail (掉尾式)    - Closing Form (收势) 3. Introduction to Mindfulness Day | 1. Taiyi Mirror Heart Chuan practice 2. Mindfulness practice: Recording Mindfulness Day | 1. Mindfulness Attitude: Non-Judging 2. The Mindfulness Journey: Bringing Mindfulness into Your Life |
| **Session 10** | 1. Review, share, and discuss personal experiences in the program 2. Closing mindfulness practice: Brief Loving Kindness Meditation | 1. Make plans for continuing practicing mindfulness and Taiyi Mirror Heart Chuan after the program | 1. Review of Taiyi Mirror Heart Chuan 2. Autobiography in Five Chapters |
